# The meaning of self-care in persons with cervical spinal cord injury in Japan: a qualitative study

**DOI:** 10.1186/1471-2377-13-115

**Published:** 2013-09-04

**Authors:** Ayako Ide-Okochi, Etsuko Tadaka, Kazumi Fujimura

**Affiliations:** 1 Graduate School of Nursing, School of Medicine, Yokohama City University, Yokohama City, Kanagawa, Japan; 2 Faculty of Health Sciences, Yamaguchi University Graduate School of Medicine, Ube City, Japan

**Keywords:** Self-care, Self-management, Rehabilitation, Illness behavior, Health promotion, Spinal cord injury

## Abstract

**Background:**

Professionals in Japan tend to regard the individual contexts of persons with spinal cord injury (SCI) as the cause of their passive participation in self-care activities or self-management. However, the meaning of self-care involves variables that interrelate with sociocultural factors. Thus, it is necessary to uncover its meaning in the perceptions of persons with cervical spinal cord injury (CSCI) in order not only to implement better rehabilitation but also to understand the sociocultural constraints that determine the injured person’s attitudes to self-care and long-term health outcomes.

**Methods:**

Semi-structured interviews with 29 CSCI participants from fourteen municipalities of Osaka, Hyogo, and Ehime prefectures were conducted. Participants contributed diverse perspectives on rehabilitation, lay-professional and family relationships, health promotion, and body conceptions. Interviews were recorded, transcribed and analyzed using the grounded theory approach to inter-relate categories and to develop theoretical constructions.

**Results:**

Four main themes emerged from the data: rehabilitation for independence in ADLs; detachment from the body and self; embodiment; and self-management. From the participants’ point of view, rehabilitation programs in Japan aim at improving body functions for ADL performance, but provide little health education. These rehabilitation values might hinder some participants from developing self-esteem for their bodies. Moreover, socially-shaped family caregivers’ active engagement in the participants’ self-care allowed many participants to entirely rely on them for care. Through embodiment, participants found that self-care was not merely a means of independence in ADLs but also of self-management to enhance health and well-being, requiring collaborative relationships with caregivers.

**Conclusion:**

Personal factors such as low motivation for self-care might be in part a reflection of social expectations of dependence for persons with CSCI. However, the shift in the meaning of self-care from ADLs to self-management implies more active participation in health care needs, shaped through social exchanges. Not only personal factors but also sociocultural factors influence the injured person’s valuation of self-care. There is a need for further research to better understand sociocultural influences on illness behaviors among persons with CSCI, so that clinical and community practice can develop accordingly.

## Background

Secondary conditions such as pressure sores, bladder infection, obesity, and pain are preventable or manageable medical complications in persons with physical disabilities [1-4]. However, once these secondary conditions occur, they cause significant interruptions in the lives of persons with spinal cord injury (SCI) [5,6]. There is a report in Japan that persons with SCI constituted about 60% of the patients at an outpatient clinic for pressure sores, and that patients did not come to the clinic for nearly a year after the onset of the sores, allowing them to reach a critical stage [7]. The authors cited above hypothesized that the reluctance of patients to look at their pressure sores was a potential risk for worsening of the sores, even if family caregivers had actually provided care in managing the sores.

Training to acquire self-care skills has been conducted as the major focus of rehabilitation programs [8-10]. Training or education in self-care includes occupational or physical therapies to learn modified ways to move body parts [9] and health education for symptom control [4]. However, rehabilitation practices have been directed to minimizing functional limitations [11] and Japan is not an exception [12,13]. One phenomenological study in Sweden found that this notion of self-care is internalized in SCI patients who were in the beginning phase of rehabilitation [9]. A study of persons after stroke in Japan also found that recapturing body motions was one of the meanings that they found in the process of reconstructing their lives [12]. The subjects of these previous studies [9,12] were persons who managed to move their body and were considered to have less severe physical dysfunctions than most persons with CSCI. Moreover, the Swedish study [9] examined perceptions of self-care by SCI patients at the very beginning of their disability (1–3 months after onset). Therefore, there is very little research which looked into the meaning of self-care in CSCI persons with a long-term perspective. It is essential to examine the self-care perceptions of those persons in order to make rehabilitation practices more oriented to social reintegration and QOL [11].

Persons with CSCI need the most extensive care among those with SCI and are inevitably involved in interactions with caregivers [14]. Therefore, CSCI persons have the greatest need to realize their responsibility in self-care and to manage attendant care services [4]. However, in Japan, collective family decision-making takes precedence over individual preferences, even today [15]. Therefore, taking care of persons with disability is viewed as the responsibility of families, who hide members with disability from society [16]. That is why, although one person with CSCI recalled that he learned maintenance of body functions during hospitalization [17], medical professionals did not expect him to manage caregivers in performing self-care. This lack of autonomy might lead to a negative self or body image [4]. In fact, previous studies [12,18] found that persons with CSCI or stroke depersonalize their body as an object of therapists or caregivers. It was not until they began social participation that patients with CSCI personalized their bodies [12,18]. Therefore, it is necessary to understand how persons with CSCI perceive their role in self-care and how these perceptions can be changed, in order to enhance their autonomy and psychosocial well-being.

However, except for a very few statements in small number of biographies of persons with CSCI [17,18], very little is known about how persons with CSCI in Japan interpret self-care. It is essential to explore the meaning of self-care among persons with CSCI from their perspective. The research questions are:

– How do persons with CSCI perceive self-care?

– What do persons with CSCI consider to be their role in performing and/or being assisted in self-care?

– Does the meaning of self-care change over time and how?

## Methods

### Grounded theory

Data were collected and analyzed based on the grounded theory approach [19]. Grounded theory means an empirical methodology in analyzing data used for the purpose of building theory from data. According to the grounded theory approach, theory is not merely distinguished from description, but also means an arrangement of categories (concepts) that are systematically interrelated to present an explanation of the phenomenon [19].

Grounded theory enables the production of thick, detailed interpretations of phenomenon [20]. This methodology was considered suitable for this study’s purpose of exploring the meaning of self-care not only through individual traits but also in social, political, and cultural conditions. Data were collected by individual interviews that were audiotaped and transcribed and by observations that were recorded in field notes.

### The sampling strategy

Theoretical sampling is one of the major principles of this methodology [20], in which data collection is continued based on concepts derived from the data, for the purpose of maximizing the opportunity not only to develop new concepts but to inter-relate existing concepts. Although in ideal theoretical sampling, the next interviewee is recruited based on developed concepts, any practical types of theoretical sampling are accepted as long as constant comparisons among concepts are made during analysis [19]. This study used a practical theoretical sampling technique to collect data in which the sampling process was conducted based on a list of potential participants.

Potential participants were recruited from personal contacts with the representatives of a nationwide self-help group and a visiting-health-service provider. In Japan, it is estimated that more than 100,000 persons suffer from SCI, and that 5000 persons incur SCI each year [21]. Of these persons, the proportion with CSCI is estimated at about 75 percent [22]. The self-help group (*zenkoku keizui sonsyosya renrakukai*) that we studied is the only group mainly consisting of persons with CSCI and it has branches in 14 of 47 prefectures in Japan [23]. Following written approval by The University of Tokyo’s Research and Ethics Committee, a query letter was sent to 31 potential participants requesting a response from those who were interested in being interviewed. The query letter included a brief description of the study and asked for participation. Potential participants were given the principal author’s office phone number and e-mail address for contact purposes. The principal investigator telephoned all potential participants to explain the study purpose and schedule a mutually convenient meeting date and time.

The information of study settings should be noted. Three branches of the nationwide self-help group were introduced as sources of potential participants. They were located in Ehime Prefecture, Hyogo Prefecture, and Osaka Prefecture. The home-visit service provider cited above is located in Ehime Prefecture and run by a private rehabilitation hospital. The characteristics of each prefecture differed in regard to medical resources for persons with traumatic SCI: there were three public rehabilitation centers for long-term hospitalization and comprehensive rehabilitation in both Osaka and Hyogo prefectures, but none in Ehime prefecture.

### The study sample

There were 2 women among the 31 potential participants who declined participation. As a result, the sample consisted of 27 men and 2 women who had sustained a traumatic cervical spinal cord injury. This was a male-dominant sample, even though the ratio of gender distribution of individuals with spinal cord injuries is about 4 men to 1 woman in Japan [24], Canada [25] and America [26]. The sample is described in Table 1.

**Table 1:** Demographic profile of the participants (N = 29)

**Characteristics**	**Number**		**Percent**
Age			
Mean ± SD (range)	48.1 ± 12.4 (26–77)		
Age at injury			
Mean ± SD (range)	30.7 ± 16.3 (14–69)		
Duration of disability			
Mean ± SD (range)	16.9 ± 9.9 (4–36)		
Level of injury			
C1	2		6.9
C3	6		20.7
C4	10		34.5
C5	6		20.7
C6	3		10.3
Unknown	2		6.9
Type of injury			
Complete	17		58.6
Incomplete	12		41.4
Marital status			
Married	10		34.5
Single (never married)	17		58.6
Divorced	2		6.9
Main caregiver			
Family members	18		62.1
Spouse		9	
Mother		6	
Father		2	
Siblings		1	
Paid caregivers	11		37.9

This study’s sample was younger in terms of age at injury, compared to 51 mean years obtained through a nation-wide epidemiological survey of CSCI persons, conducted in the early 1990s [22]. Moreover, this study’s participants had more severe type of injury than sample in the nation-wide study cited above, in that the complete type accounted for 21.2% [22]. Family members consisted of 62.1% of main caregivers, which was similar to the ratio of 67% in a study of ventilator-dependent CSCI persons in Japan [27].

### Interview

A semi-structured interview was used to explore the meaning of self-care from the insider’s perspective. Although these questions (shown in Table 2) were guided in a semi-structured interview protocol, questions were increasingly focused and detailed in subsequent interviews. This developing interview protocol meant that the purpose of theoretical sampling was achieved in that hypotheses from the emerging theory were tested [19,28].

**Table 2:** Interview guide

**Main question**	**Topics**
“What do you think of self-care in your life as a person with CSCI?”	Experiences of institutionalized rehabilitation
	Conditions of subjective health and care service use
	What participants have done as self-care activities
	Who manage self-care needs and relationships between caregivers
	Body/self image
	Conditions of social participation and their meaning

All interviews, with one exception, were conducted in the participant’s home from April to August in 2009. Only one interview was conducted at a private room of a workshop for persons with disabilities. The principal researcher observed participant’s interactions with caregivers and jotted down those interactions and nonverbal information during interviews.

### Analysis

It is one of the major characteristics of qualitative studies that the processes of data collection and analysis overlap and that researchers can launch analysis after the first interview [19]. Analysis used in the grounded theory approach involves open coding, axial coding and selective coding to develop analytic categories. Open coding is the process of dividing data into meaningful parts (codes) to make categories so that similar codes are gathered and constantly compared to develop more abstract concepts. Axial coding is used to inter-relate categories to maximize the potential of developing theories. Selective coding is used to develop core categories that integrate whole categories and provide cohesive explanations of phenomena. Data were considered saturated when no more codes could be identified, existing categories were coherent, and there were enough variations to explain categories.

### Trustworthiness of the data and interpretations

Trustworthiness is the equivalent of validity in quantitative research [29]. Writing decision trails in which the methods of data collection and analysis are presented is important so that another researcher can check the trustworthiness of the whole process of study [30]. Guba and Lincoln [29] presented four concepts for checking trustworthiness and decision trails in qualitative research: credibility, transferability, dependability, and confirmability. Credibility is demonstrated through a) member check; b) peer debriefing; c) prolonged involvement; d) persistent observation; and e) triangulation [30]. Five participants checked a summary of emergent themes and research transcripts and assured themselves that the interpretation was true to their perceptions. An expert in qualitative research checked that the data and interpretations were coherent and audited the study process. The principal researcher had known 18 participants through personal contacts prior to this study and even now has plentiful opportunities for observing persons with CSCI through professional and personal contacts. Moreover, multiple sources for recruiting participants assured the variations in data that are the rationale for triangulation.

Transferability was accomplished by providing a description of study samples and models of this study to demonstrate generalizability of study results. Dependability was ensured by having independent analysts review the data and contest the themes. Confirmability was accomplished by providing decision trails between data and interpretation.

### Ethical considerations

This study was a part of doctoral dissertation at The University of Tokyo. Research approval was obtained from The University of Tokyo’s Research and Ethics Committee. All participants were informed of the objective and design of the study and a written consent was received from the participants for interviews and they were free to leave the interview if they wished.

## Results

Four main themes emerged from the data. These themes reveal how participants interpreted the meaning of self-care and what factors influenced the construction of its interpretation. The four themes were: a) rehabilitation for independence in ADLs; b) detachment from the body and self; c) embodiment; and d) self-management. These themes and their subcategories are presented in Table 3.

**Table 3:** Themes and subcategories

**Themes**	**Subcategories**
Rehabilitation for independence in ADLs	Regaining independence in ADLs
	Having faint image of what I can
Detachment from the body and self	Disvaluing the body
	Intended obedience
Embodiment	Knowing the new body
	Regaining self-efficacy
	Realizing responsibility
Self-management	Internal locus of control over daily tasks
	Promoting health and well-being
	Involving social interactions

### Theme 1: rehabilitation for independence in ADLs

#### Regaining independence in ADLs

Participants demonstrated that rehabilitation as a continuum of acute care after the injury was focused on the achievement of independence in ADLs. Participants who could perform a part of their ADLs by themselves stressed how they struggled with learning new ways of moving their body parts. They viewed self-care as performing ADLs by themselves as much as possible.

“For the purpose of doing my things by myself, I did rehabilitation. At first, paid attendants were present. For the ultimate goal, I wanted to stand up and use the bathroom by myself.”

#### Having faint image of what I can

Some participants said that they had been bedridden and could not move their body parts as they could before the injury. Although there were some participants who understood rehabilitation goals such as standing upright by themselves, some participants admitted that they were suspicious of what they could achieve as a rehabilitation goal, unless they could transfer themselves at will. A few participants recalled that they had imagined that before rehabilitation they could achieve something other than being bedridden. In reality, not a few participants stated that they found that they still could not move their body parts satisfactorily and therefore could not perform ADLs as they could before the injury.

A few participants stated that they so often complained about unimportant things during hospitalization. They had admitted that they lacked the motivation to voluntarily participate rehabilitation sessions. A participant recalled that he did not dare to think he could eat by himself by using a spoon, while he was admitted to an orthopedic surgery unit where most of impatiens was both persons with after effect of stroke and amputated persons because of bone tumor; therefore, he could not meet a person with CSCI.

“There were none who had had a same type of disability as mine. Therefore, my motivation to tackle rehabilitation hardly increased, that I recall now.”

### Theme 2: detachment from the body and self

#### Disvaluing the body

Some participants expressed negative feelings about their bodies. They disvalued their bodies in terms of body image and of their limited abilities to accomplish body actions. One participant with a ventilator stated that her body was not healthy at all because she could not walk, talk, and have an ordinary life as a non-disabled person would.

There were other participants who had not actively taken care of their bodies because they stated that they did not like their bodies with their disabilities.

“…when I was unable to take an interest in my body, I had high fevers. Well, maybe I could not have any pride in my body.”

Besides low interest in their bodies, some participants stated that they were ashamed of their impaired body. Some of them were keen on strangers’ gaze therefore they once had been socially withdrawn and underestimated the necessity to see a doctor regularly. Furthermore, a participant, who had a history of social withdrawal, said that he could not ask strangers to help his eating because he felt ashamed of his impaired body and therefore he did not want to hurt his pride.

#### Intended obedience

Participants stressed that it was not they but their family caregivers who made the decisions about day-to-day regimens and the timing of giving medicine while participants lived with family members.

“…as for antibiotics, mother sees my urine without my request, and I assume she decides to give [antibiotics] or not. I only open my mouth and take a tablet set by my mother, so I can’t see those”

Some participants said that they had suffered a mental conflict between awareness of self-responsibility for their injury and their physical conditions that assistance was necessary. Mothers had been a main caregiver in a case of 18 participants and they stated that mothers had become old and ailing. In perceiving that they had caused their mothers a trouble, some participants tried not to call mothers even when they had something to ask for assistance of self-care.

Some participants stated that they had to put up with the situations in that family caregiver preferences took precedence over those of the participants because they were taken care of by family members. In their perception, it was morally justified that they were not assertive in making a decision how to perform their daily tasks, even when they had other needs than family caregivers’.

“Once I came home (from the rehabilitation facility), I had to live without making a burden on the family, for them to live as easily as possible. Even if I want to ride on a wheelchair, I can put up with it.”

### Theme 3: embodiment

#### Knowing the new body

Participants stated that their body could not move; besides, they mentioned that they could not use their body parts to perform their self-care. A participant who was used to work for a wholesale company of pharmaceutics said that he knew that his body would not move for life in the first day of his hospitalization immediately after the injury. The other participant stated that it was not until he began to doubt the potential recovery of his body that he found a surgery which he longed for did not bring any substantive change to his paralyzed body.

“There had no change occurred after the surgery. The only change had occurred at my thumbs. These were solely and only changes I found. I could not regain even any sensations. Then, I began to think my body’s conditions would last forever.”

#### Regaining self-efficacy

Participants stated that they understood what physiological functions were possible and how they could control symptoms due to the injury. Participants stated that encounters with persons with SCI or CSCI have helped them to know what their bodies could achieve. One participant who said that he was not motivated for rehabilitation recalled that seeing other CSCI persons during secondary hospitalizations let him realize his possibilities.

“At that time, I had not imagined that I could eat meals with my hands. When I was admitted to that ward, I found that everyone was accustomed to using a variety of instruments during hospitalization. By seeing that, I considered that I could do it…”

Another participant stated that he could play a role in aiding urination by pressing his stomach when he lay on a bed and it spared his mother from having to care for his urine throughout the night.

#### Realizing responsibility

Except for one, participants had to ask family members to rebuild, modify, or purchase a house or change their apartment because of the injury. In another instance, a mother had to quit her job to care for her child. Participants stated that they had realized their responsibilities for maintaining health because if they had complications they placed additional burdens on family caregivers or anyone else concerned.

“…if my conditions get worse, I will struggle of course, but I think of my family beforehand, well, my household will be in a mess, I rather care for it. Because of that, I think I have to keep fit”

Some participants stated that it was until their mother had been hospitalized or passed away that they noticed that they relied mostly on their mother for nearly everything in performing daily tasks such as transfer, dressing and excretion. A participant recalled with regret that he knew his mother’s dedication to caregiving enabled his active social life as a social worker only after his mother had passed away after stroke.

“My life had went satisfactory until then, but suddenly my mother broke down…there are family members that persons with disability can resort to. That is the principle of caregiving in Japanese society. I thought I had to get out of that custom.”

### Theme 4: self-management

#### Internal locus of control over daily tasks

Some participants stressed the importance of their own management of self-care. Even though it was actually a caregiver that made most of participants bathe themselves; dress and groom before going out; and excrete wastes, some participants considered that they should have known options of self-care in that they themselves could verify their bodily condition. A participant mentioned that enabling him to see his stool was one of the merits that evacuation with an artificial stoma possessed, when he compared the merits between evacuation with an artificial stoma and assisted evacuation with unaccustomed nurse’s fingers.

Some participants asserted that they would like to live a life in that they could manage someone other than family caregivers. These participants considered that employing paid caregivers made them perform assisted self-care as satisfactory as they desired, because they hardly had a feeling of guilt in asking to do trivial tasks for them, while they had hesitation to ask family caregivers.

“I do not have to worry about home-helpers’ response than my parents’. Because my parents stand by me while they live own life, when I sensed that they want to bathe themselves this very minute, I will not ask them to assist me even when I actually want to ask them.”

#### Promoting health and well-being

Based on the perception that maintenance of their community lives was important, participants actively performed self-care or self-management to maintain their health. Participants assumed that they might lose their current lives, which had reached a state of normalcy after struggles with the injury, if they were hospitalized again. Therefore, health maintenance was considered essential in participants’ lives.

“…one important thing in my self-care is health: that means how much I know potential risks by myself. …I think I can do something to maintain my current life. For that, health is one thing.”

As participants sought comfortable, satisfying ways of controlling day-to-day health necessities, they paid more attention to QOL issues. Under the influence of the Independent Living movement, some participants who belonged to self-help groups stated that enhancing QOL was the key concept in deciding to choose the method of bowel management from aided evacuation with professionals’ hands to evacuation with an artificial stoma. Participants stated that the merit of evacuating with an artificial stoma was that it enabled them to be more socially active by making them free from concerns about failures in bowel management during meetings.

“…an artificial stoma, I suppose, not only in dealing with evacuation, but it leads to mental steadiness…when I think of QOL, compared with evacuation with home-helpers’ hands, it is rather faster and everyone can take care of it.”

#### Involving social interactions

In performing their own self-care, participants demonstrated that others’ involvement was necessary. Although once some participants had suffered from others’ control over their care, after they understood the necessity of aides because of their impaired bodies, they accepted that the help of aides was indispensable in their lives.

“…some things I cannot do by myself. Those things, this person performs instead of me.”

Through the perception of needing someone’s help, participants were able to instruct personal assistants or home helpers about what help they wanted. However, at the same time participants tried to build collaborative relationships with personal assistants or home helpers by letting them understand their sense of life values.

In addition to social interactions with personal assistants, participants demonstrated their collaborative relationships with family caregivers in the long-term maintenance of their health after discharge. Some participants cooperated with their families for achieving long-term health for shared social goals and their preferences were taken into consideration when being provided aid.

## Discussion

### Two meanings of self-care and the shift of its meanings

This is the first research in Japan that explored the meaning of self-care in persons with CSCI. This study suggests that there are two meanings: rehabilitation for independence in ADLs and self-management. This is important because the meaning of self-care has often been viewed by both patients and professionals as recapturing body movements in ADLs [8-13]. Especially in Japan, the aspect of self-management in the notion of self-care has received little attention because medical professionals tend to regard the word self-management as relevant to chronic diseases such as diabetes [31]. Under these medical circumstances, the finding of the notion of self-management in participants’ perception of self-care has importance for making rehabilitation practices more patient-centered.

We have also implied that the enlargement in the meaning of self-care from ADL skill trainings to the management of health can have an impact on QOL. This shift in the focus of self-care connotes various aspects of CSCI: one is trauma that needs acute care and the other is the chronic neurological dysfunction that needs life-long health management. The strength of this study is the inclusion of participants who had a longer duration of disability compared with the participants of other qualitative studies [9,25,26]. Moreover, this study confirmed that participants could become keen on their QOL. QOL is often used as one of outcomes of long-term adjustment in persons with SCI [11]. Here, the inclusion of participants with relatively long durations of disability has helped to reveal that participants’ notion of health had changed from survival-oriented or ADL-oriented to QOL-oriented [32].

The finding of this shift in participants’ main focus of self-care implies the necessity to rethink the role of rehabilitation. We hypothesize that rehabilitation practices have not effectively conveyed the message that the loss of movement and sensations of the head and neck area does not necessarily mean the loss of the ability to manage health care needs and to instruct attendants [12,13,18]. As a result, when persons with physical disability find that independence in ADLs has not been achieved, they sometimes lose their sense of meaning in life and become socially withdrawn [12]. It is essential for professionals and health policy makers to better understand the health care needs and rehabilitation goals of persons with CSCI. Our study has provided some clues to help persons with CSCI and caregivers to share long-term goals.

### Embodiment and the need of psychosocial support

This study showed there were the two themes of *disvaluing the body* and of *embodiment* in the process of developing notions of self-care. The theme *detachment from the body and self* probably parallels the notion of self-neglect, which is seen as the cause of secondary health problems such as pressure sores and urinary tract infections [7,33]. However, the findings of this study imply that some participants’ low levels of concern for the body and health maintenance were also social constructions. It might be hypothesized that medical practices in Japan that highly value the potential for improvement from disability [12,13,17] had affected in some ways how participants interpret their bodies and what value they put on themselves. The influence of professional attitudes to the disabled person’s body is probably so significant that injured persons could easily develop self-neglect.

The results of this study imply that some family caregivers’ involvement with participants’ self-care has been so intense that participants could entirely rely on family caregivers without taking any interest in their own body mechanisms. Some participants’ passivity in family relationships resonate with previous studies showing that family members often control persons with disability [12,17,18] because taking care of disabled family members is considered as a family obligation [16]. This study further implies that some participants’ passivity in self-care has been chosen in order to live in harmony with family members, given that social norms expect sick persons to fulfill the ideal sick role by seeking to cooperate with caregivers [34]. This theme of *intended obedience* shows the difference in the socially admitted patient’s role between America [35] and Japan [15,36]. It is a social obligation even for persons with severe physical disabilities to live independently in America. However, in Japan [15,36], it has been considered as a virtue of patients that they do not insist on their own opinion and let family members make important decisions instead of the patients themselves. This study shows how it is important to let persons with CSCI in Japan understand that obedience is not a suitable patient role.

As participants learned to know their new bodies and what they could achieve, they realized that it was not caregivers or professionals but they who had to take on their self-care to control symptoms. The finding of this process, termed *embodiment*, is considered important because previous research in Japan [7] had not found what factors lay in the differences between active patients and passive patients in respect of performing self-care. We found that having a positive body image through *knowing the new body* and *regaining self-efficacy* might influence participants’ motivation in self-care. Previous research has paid attention to the body image of persons with SCI as a potential factor in social participation [25,26]. This study found that having a positive body image is also important in the personalization of self-care in that individuals mature over time and come to see themselves as self-care agents and to exercise responsibilities to engage in their self-care [37].

This is why providing psychosocial support in rehabilitation is considered essential for persons with CSCI to actively participate in their self-care. Peers with CSCI were especially helpful for participants in seeking answers about what they could do and what responsibility they had in their self-care and lives. A previous study of coping in persons with SCI demonstrated that through interactions with other SCI patients, participants could develop a positive appraisal that the consequences of SCI were controllable [37]. Positive peer influence on psychological adjustment is likely to be encouraged by the results of this study, which imply that interactions with other persons with CSCI worked as psychosocial supports that complemented the narrow niche of ADL-laden rehabilitation programs [11].

Unfortunately, persons with traumatic CSCI did not have equal opportunities to see and communicate with peers. Rather, the opportunities to learn from peers were obtained by coincidence, as were the encounters with knowledgeable professionals [4,38], because professionals usually do not inform patients with CSCI of self-help groups [18]. Again, professionals and health policy makers should take into account that psychosocial supports are required for persons with CSCI so that they can understand their capabilities to achieve well-being and to realize their self-care agency.

### Limitations of this study

There are some theoretical limitations in this study. First, it is beyond doubt that personal factors such as readiness to learn or seek information [2], patient communicative health literacy [39], gender [28], general former background [40], and preference for paternalistic patient-physician relationships [15] have impacts on participants’ perceptions of self-care and their consequent behaviors to tackle health care necessities [4]. There is also a need to study the influences of these personal factors on an individual’s health state.

This study has deficits in taking the influences of the shift in health and welfare policies into account. With respect to the availability of home helpers, it is likely that there were not enough of them for participants to use as a complement or an equivalent to family caregivers because the deployment of caregivers (home helpers) had been aimed at caring for the elderly since the 1960s [41]; there were practical limitations on the length of available time with paid caregivers that municipalities could approve and especially individuals living with family caregivers were approved for shorter lengths of time with paid caregivers [18,41]. Although securing 24-hour-aid was approved in 1993 in Tokyo, most municipalities across Japan have not achieved it [41]. The influence of sociocultural factors might shift through time, in accordance with changes of health and welfare policies and social norms [42]. There is a need in future studies to examine the influence of policy changes on individual perceptions of health and illness behaviors.

The sample of this study may not represent all CSCI population in Japan, considering younger age at injury and severer type of injury than the sample of previous nation-wide survey in Japan [22]. There were also limitations derived from the unavailability of enough female participants. This study’s sample was more male-dominant than the general gender ratio of persons with SCI [24-26]. Therefore, specific influences of gender on the perception of self-care were not fully investigated, although females with SCI perceived vulnerability in male-dominant rehabilitation settings [28]. Perceptions of self-care in women with SCI should be further explored.

## Conclusions

Our qualitative research provides an overview of participants’ perceptions of self-care where personal profiles and social systems interact to shape the meaning of self-care. Participants mentioned self-neglect, which professionals had assumed to be the reason for passive participation in self-care and consequent ill-health. However, minimal concern for self-care might be in part a social construction in that professionals value independence of ADLs and in turn expect severely limited participants to rely on their family members. Moreover, the process of participants learning the importance of self-management was also a social representation shaped through social exchanges. Disability is a dynamic process in which person-environment interactions take place. The findings of this study illuminated the need to better understand both personal and social factors to effectively intervene in the study population’s contexts to promote health and QOL.

## Competing interests

The authors declare that they have no competing interests.

## Authors’ contributions

AI designed the study, collected and analyzed data, and drafted the manuscript. ET helped to conceive the study and helped to review the manuscript. KF helped to review the manuscript. All authors read and approved the final manuscript.

## Pre-publication history

The pre-publication history for this paper can be accessed here:

http://www.biomedcentral.com/1471-2377/13/115/prepub
